# Can genetics reveal the causes and consequences of educational attainment?

**DOI:** 10.1111/rssa.12543

**Published:** 2019-11-24

**Authors:** Marcus Munafò, Neil M. Davies, George Davey Smith

**Affiliations:** ^1^ University of Bristol UK

**Keywords:** Causal inference, Education, Genetic structure, Mendelian randomization

## Abstract

There is an extensive literature on the causes of educational inequalities, and the life course consequences of educational attainment. Mendelian randomization, where genetic variants associated with exposures of interest are used as proxies for those exposures, often within an instrumental variables framework, has proven highly effective at elucidating the causal effects of several risk factors in the biomedical sciences. We discuss the potential for this approach to be used in the context of social and socio‐economic exposures and outcomes, such as educational attainment.

Socio‐economic inequalities in cognitive and social skills are apparent before children even start school (Blanden *et al*., 2007; Kautz *et al*., 2014). These differences are large and pervasive, and their effects persist over the life course, subsequently crystallizing in differences in educational attainment. The concept ‘educational attainment’ is intuitive and seemingly straightforward, but its measurement is not. There is variation in the number of years that are spent in formal education, but also differences in attainment between those who spend a similar number of years in education (e.g. the number and grade of secondary school qualifications), and even more fine‐grained differences between those who achieve similar levels of attainment (e.g. similar university grades at more or less prestigious universities). This is a particular challenge in large‐scale genomewide association studies, which typically combine data from multiple samples, drawn from different birth cohorts, different educational systems, and so on. The solution to this data harmonization problem is to rely on a simple, universal but relatively crude metric—the number of years spent in education. In some education systems this measure may be misleading, e.g. in education systems that routinely hold back students who have failed a grade.

There is a broad literature exploring different potential causes of educational inequalities at different ages. This includes studies in economics and the social sciences (e.g. economics, policy studies, sociology and education), and biomedical sciences (e.g. psychology, epidemiology, genetics and epigenetics) (Eccles, 2005; Feinstein, 2003; Jerrim and Vignoles, 2013; Krapohl and Plomin, 2016; Little *et al*., 2019). These studies have explored a large set of potential inputs into educational attainment, such as the *in utero* environment, and parental, family, school and teacher factors (Burgess, 2016; Karlsson Linnér *et al*., 2017; Lee *et al*., 2018; Slater *et al*., 2012). However, determining causal links from these factors to educational attainment is challenging. Researchers have used a variety of approaches to estimate the contributions to education of the child's cognitive abilities and psychological traits (or essential life skills—often called ‘non‐cognitive traits’) as well as parental inputs of time and money (Smithers *et al*., 2018).

Another large interdisciplinary literature explores the life course consequences of educational attainment, such as for health and wellbeing, earnings, life satisfaction, fertility, parenting and partnership formation. From the original work of Grossman (1972) and others (e.g. Mincer (1974) and Becker (1975)) onwards, economists have estimated the causal effect of education on earnings and other outcomes. Many different approaches have been used to estimate causal effects of education, including instrumental variables, regression discontinuity designs and comparing outcomes between monozygotic and dizygotic twins. This literature suggests that schooling causes a substantial increase in individual earnings (see for example Card (2001) and Heckman *et al*. (2006)), as well as a reduction in workplace injury rates, unemployment, welfare receipt and quicker entry into the labour market (Oreopoulos and Salvanes, 2011). There is also a considerable literature showing evidence of causal linkages between education and health and other non‐pecuniary outcomes (Grossman, 2006; Oreopoulos and Salvanes, 2011). However, there is also uncertainty regarding whether these differences are due to a causal effect of education. Many studies suggest that differences in educational attainment are large and important for later life outcomes, and estimated causal effects on some outcomes are relatively robust across different samples and empirical approaches (e.g. the effect of education on earnings), but there is conflicting evidence on the effects of education on later life health and mortality (Albouy and Lequien, 2009; Clark and Royer, 2013; Davies *et al*., 2018a; Galama *et al*., 2018; Lleras‐Muney, 2005; van Kippersluis *et al*., 2011). These differences in health outcomes between educated and less educated groups could be due to pre‐existing differences that occur before education (e.g. in diet, physical activity and socio‐economic position).

Recent insights into the genetic influences on a range of socio‐economic, biological, behavioural and health phenotypes now enable us to apply Mendelian randomization to these questions (Davey Smith and Ebrahim, 2003; Davies *et al*., 2018b). This is an instrumental variable approach, which uses genetic variants (typically single‐nucleotide polymorphisms (SNPs), as proxies for potentially modifiable exposures of interest). It requires SNPs that

(a) are associated with the exposure of interest,
(b) share no causes with the outcome of interest and
(c) do not directly affect the outcome via mechanisms that are not mediated via the exposure (Fig. 1(a)).

**Figure 1:**
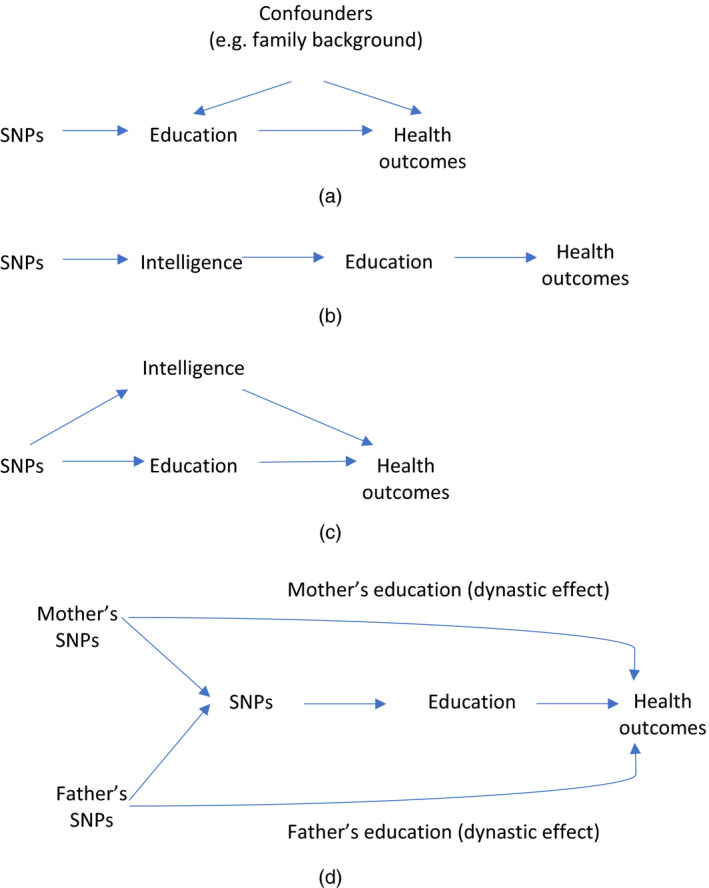
Causal diagram indicating possible relationships between education and later health outcomes, and how Mendelian randomization can be informative: (a) genetic variants as instruments for education; (b) vertical pleiotropy; (c) horizontal pleiotropy; (d) dynastic effects

The first assumption is directly testable. The second assumption is likely to hold in many applications because of the random inheritance of alleles from parents at conception, and it can be falsified by examining the association of SNPs of interest with measured confounders. The third assumption is not directly testable, but there are an increasing number of instrumental variable estimators that are robust to violations of this assumption (Bowden *et al*., 2016; Davies *et al*., 2018b; DiPrete *et al*., 2018; Hartwig *et al*., 2017; Hemani *et al*., 2018; Windmeijer *et al*., 2019). Furthermore, it is important to distinguish between vertical pleiotropy (Fig. 1(b)), where a variant affects a phenotype such as cognition which goes on to affect education and in turn a health outcome, and horizontal pleiotropy (Fig. 1(c)), for example, where a variant affects health outcomes without being mediated via education. Only horizontal pleiotropy causes bias in a Mendelian randomization analysis and is a threat to both the second and the third assumptions described above. So Mendelian randomization will only be biased if SNPs affect an outcome, and education does not fully mediate the effects of the SNPs. It is worth noting that horizontal pleiotropy is likely to be a particular concern in the context of complex and distal phenotypes such as educational attainment, where the effects of genetic variants are likely to operate via a range of biological, behavioural and social pathways to influence the outcome.

Other threats to Mendelian randomization studies that are particularly relevant in the context of educational research include assortative mating and dynastic effects. Assortative mating occurs when individuals that are phenotypically similar—and therefore genotypically similar—are more likely to have offspring together (Hartwig *et al*., 2018). Dynastic effects occur, for example, when the parents’ education‐associated SNPs affect their offspring's outcomes because of their effect on the parents’ own educational attainment (Fig. 1(d)). Both assortative mating and dynastic effects can cause bias and false positive findings in Mendelian randomization studies of the effect of education. Cohort studies that include genetic information on both parents and children or samples of siblings enable these potential threats to be investigated and overcome. For example, this enables examination of the effects of parental transmitted *versus* non‐transmitted alleles on offspring outcomes. Indeed, the ability to investigate transmitted and non‐transmitted alleles in principle enables us to examine effects of parental education *versus* offspring education on a range of outcomes—something that Mendelian randomization using data from unrelated individuals cannot do because of the correlation between offspring and parental genotype.

Mendelian randomization has proven highly effective at elucidating the causal effects of several risk factors in the biomedical sciences, as well as providing an indication of when risk factors may not affect an outcome. This approach has several potential advantages that are relevant to education research, including robustness to measurement error, reverse causation, endogeneity and confounding, and provides a strong basis for causal inference, particularly when results from this approach are triangulated with those from other approaches that rely on differing assumptions. It has successfully predicted the findings from several randomized controlled trials before the trials were completed and is transforming how drug targets are validated (Walker *et al*., 2017). The approach has also clarified the health consequences of behaviours (such as alcohol use, smoking, physical activity and obesity) and the relationship between psychosocial indicators such as a sense of wellbeing and health (Wootton *et al*., 2018). These techniques can potentially be used to address critical questions relating to the determinants and consequences of educational attainment. Researchers have conducted genomewide association studies of educational attainment by using very large samples of data (*N*=1.1 million) from around the world (Lee *et al*., 2018). These studies reported 1271 SNPs associated with educational attainment at the genomewide significance threshold of *p*<5×10^−8^. Across the entire genome, common SNPs explain 11–13% of the variation in educational attainment, meaning that they strongly predict educational attainment and can provide instruments for use in other samples. Recently this approach has been used to demonstrate a substantial protective effect of education on coronary heart disease, with partial mediation of these effects being through health‐related behaviours such as smoking and obesity‐related traits (Tillmann *et al*., 2017). These links can be further interrogated through multivariable Mendelian randomization, e.g. demonstrating that the causal effect of education on smoking does not simply reflect cognitive ability (Sanderson *et al*., 2019a). It has been used to study antenatal maternal and paternal influences (Davey Smith, 2008; Lawlor *et al*., 2017), suggesting that maternal alcohol use during pregnancy has detrimental effects on educational outcomes, even when drinking is in the light to moderate range and without binge drinking (von Hinke Kessler Scholder *et al*., 2014; Zuccolo *et al*., 2013).

A striking example of how genetic studies can identify the direction of causation is provided by evidence from recent studies investigating the relationship between educational attainment and myopia (Mountjoy *et al*., 2018). It is possible that myopia could lead to worse educational attainment, e.g. if pupils with uncorrected myopia cannot read the blackboard, or improved attainment, e.g. if myopia leads to ‘bookish’ children who spend more time reading and therefore learn more. However, there is little evidence that SNPs that are associated with myopia at genomewide levels (Pickrell *et al*., 2016) associate with educational attainment. Conversely, there is evidence that SNPs that associate with educational attainment at genomewide levels (Okbay *et al*., 2016) also associate with myopia. These findings suggest that something related to the educational environment influences rates of myopia in the population, but that myopia does not impact on educational outcomes, at least in the context where glasses are an effective and ubiquitous intervention. More research is needed to determine which social or policy interventions can help to break the effects of education on myopia. Further evidence that the effects of education on myopia are likely to be due to an aspect of the environment is provided by changes to rates of myopia in certain east Asian countries, which have increased as the intensity of the education systems in those countries has increased. The underlying distribution of genetic variation in the population cannot have changed sufficiently quickly to explain these changes. The direction of causation between myopia and education has been the subject of research for many years, but other than through randomized controlled trials there is no way to obtain definitive causal evidence of the effects of the educational environment on myopia. However, although a recent randomized controlled trial provided evidence that interventions during education can reduce myopia (He *et al*., 2015), these are typically challenging in this context, often expensive, time consuming, underpowered, unrepresentative and not always possible.

Genetics, through the application of Mendelian randomization methods, can therefore provide a valuable source of evidence to address questions regarding the causes and consequences of educational attainment. Its underlying assumptions and potential sources of bias are potentially quite different from other current methods, so it is likely to be most powerful when used in combination with these other non‐genetic approaches, such as natural experiments, within a triangulation framework (Lawlor *et al*., 2016). The increasing availability of genomewide association studies summary data, and the development of multiple methods for interrogating these, offers considerable scope for rapidly and cost‐effectively generating valuable causal evidence. Mendelian randomization can also be used to identify intermediate pathways (i.e. the specific aspects of modern educational practices). This requires genomewide association studies of potential mediators (Sanderson *et al*., 2019b). However, as with any methods, there are limitations. In particular, horizontal pleiotropy is a threat to the assumptions of Mendelian randomization, and these assumptions can either not be tested, or tested only imperfectly. This necessitates the use of multiple methods, including a range of pleiotropy robust and within‐family methods with different assumptions and sources of bias (Pingault *et al*., 2018). Although the field continues to evolve, guidelines now exist for the reporting of Mendelian randomization studies (Davies *et al*., 2018b). Moreover, interpretation may need to be cautious—it is highly likely that true causal pathways to and from educational attainment are context dependent. In the past higher educational attainment was associated with higher body mass index in the UK, whereas now the opposite is true (Davey Smith, 2003). Similarly, in many parts of India higher educational attainment is associated with high body mass index today (Subramanian *et al*., 2013). For upstream causes like education, causation will be context dependent but is no less ‘causal’ because of this. Identifying what are the causes, correlates and consequences of educational attainment (including both positive and negative outcomes) is clearly of profound societal importance and policy relevance. Determining the direction of causality is notoriously difficult for education studies, but evidence from genomewide association studies, and the application of this knowledge in Mendelian randomization, provides a powerful new tool from a perhaps unexpected quarter.
